# Weight Status, Physical Activity, and Depression in Korean Older Adults

**DOI:** 10.2188/jea.JE20170083

**Published:** 2018-06-05

**Authors:** Jinkyung Cho, Youngyun Jin, Hyunsik Kang

**Affiliations:** College of Sport Science, Sungkyunkwan University, Suwon, Republic of Korea

**Keywords:** gerontology, depression, physical activity, body mass index

## Abstract

**Background:**

This study aimed to explore the associations between weight status, physical activity, and depression in Korean older adults.

**Methods:**

We used the baseline data drawn from the 2008 baseline survey utilized in the Living Profiles of Older People Survey, comprised of 15,146 community-dwelling older people (42.6% men and 57.4% women) aged 60 years and older residing in the Republic of Korea. After excluding respondents with missing data on height, weight, and physical activity (PA), data on 10,197 samples (43.3% men and 56.7% women) were analyzed in this study.

**Results:**

Underweight and completely inactive individuals had poorer sociodemographic and health behavioral characteristics and increased risks of late-life depression compared with normal weight and sufficiently active individuals, respectively. In terms of the aerobic PA guidelines, completely inactive individuals had a significantly higher risk of late-life depression (odds ratio 1.730; 95% confidence interval, 1.412–2.120) compared with sufficiently active individuals, even after adjustments for age, education, household income, night sleeping, living status, marital status, smoking, number of comorbidities, nutritional status, self-reported health status, and cognitive performance as covariates. In addition, those who did not meet the PA guidelines and were underweight or overweight/obese were more likely to have late-life depression compared to those who were active and normal weight.

**Conclusions:**

The current findings of the study suggest that modifiable, lifestyle risk factors, such as physical inactivity, underweight, and overweight/obesity, are positively associated with late-life depression in Korean older adults.

## INTRODUCTION

Thanks to medical, social, and economic advances over diseases, the global population aged 60 years or older has increased in nearly all countries. For the same reasons, Korea is moving toward an aged society at the fastest pace in the world. In 2010, Koreans aged 65 years and older reached 11% in the population, and the number is projected to increase to 24.3% in 2030 and reach 37.4% in 2050.^1^ While the majority of older adults remain mentally healthy, many of them are at risk of developing mental disorders, such as depression.^2^ Depression is projected to be the second leading cause of disability worldwide in 2020.^3^ Depressive symptoms can be acute or chronic, are often recurrent, and can considerably impair an individual’s ability to carry out activities of daily living.^4^

Because of its devastating consequences, late-life depression is a serious public health concern^5^ and is associated with increased morbidity and mortality,^6^ greater cognitive and functional decline,^7^ and dementia.^8^ Despite its prevalence and significance, however, late-life depression is under-recognized and undertreated due to its complicated etiologies and can be mistaken as a normal part of aging. Risk factors leading to the development of late-life depression include female sex, disability, developing a new medical illness, poor health status, prior depression, poor self-perceived health, bereavement, sleep disturbances, somatic illness, cognitive and/or functional impairment, and lack or loss of close social contacts.^9^ Additionally, advancing age is also considered a risk factor for depression, in part because of naturally occurring changes in lifestyle factors, such as obesity and decrease in physical activity.^10^

Weight status, such as obesity, is significantly associated with increased risk for chronic diseases, including type 2 diabetes and cardiovascular disease, and can lead to premature death. In addition to sharing those common health complications, studies have shown increased risk of depression in obese individuals and increased risk of obesity in depressed individuals, implying reciprocal relationships between the causes of depression and obesity.^11^ However, the relationship between obesity and depression at baseline is controversial, with previous studies reporting positive,^12^ null,^12^ negative,^13^ or even U-shaped associations.^14^ The inconsistency in the association between weight status and depression might be partially due to individual variations in the potentially modifiable risk factors, such as low socioeconomic status, loss of spouse, living alone, chronic co-morbidities, cognitive impairment, nutritional status, smoking, drinking, and others.^9^ Thus, the association between weight status and late-life depression remains to be confirmed, while considering as many potential covariates as possible.

A growing body of work has reported that regular physical activity (PA) is significantly associated with lower incidence of depressive and anxiety symptoms, reduced stress, and improved mood.^15^ By analyzing data obtained from the National Health and Nutrition Examination Survey 2005–2006, Lee et al^16^ found that accelerometer-based active/high active groups (7,500 steps per day) had a significantly lower risk of depressive symptoms compared to sedentary groups (5,000 steps per day). In a representative sample of Spanish adults, Romo-Perez et al^17^ showed that individuals who met the World Health Organization (WHO) guidelines for regular walking (≥150 minutes/week) had positive self-rated health and better body mass index (BMI) profile than those who did not meet the guidelines. In a 3-year prospective cohort study, Yoshida et al^18^ assessed the association between habitual PA and depressive symptoms in Japanese older adults, and they found that sustained PA was an independent predictor for the incidence of depressive symptoms. Similarly, Ku et al^19^ examined the relationships between changes in PA and depressive symptoms in a population-based sample of Taiwanese older adults over an 11-year period. This study found that baseline PA was associated with changes in depressive symptoms. By reviewing previous cross-sectional studies, Teychenne et al^20^ found that sedentary behavior was positively associated with anxiety risk, and the associations ranged from weak and moderate. By analyzing randomized clinical trial studies, Stonerock et al^21^ showed that regular exercise might be a useful and effective means for the treatment of anxiety. Together, the previous findings suggest that PA is significantly and independently associated with depression in older adults.

Although a number of previous studies have reported a significant association between PA and depression, potential modulators, such as sociodemographic and health behavioral risk factors, are not well considered in determining the association between PA and depression in older adults. In addition, little is known about the specific components of PA that are important, particularly the dose or domain of PA that might confer mental health benefits for older persons. Thus, gaining insight into the associations between the modifiable lifestyle factors and depression will contribute to the development of improved options for the prevention and/or treatment of mental disorders among Korean older individuals. In a representative sample of Korean older adults, this study investigated 1) the associations between weight status, PA, and depression and 2) the modulating effects of sociodemographic and health behavioral risk factors on the associations between the conditions.

## METHODS

### Study sample (data source)

Data were drawn from the 2008 baseline survey utilized in the Living Profiles of Older People Survey (LPOPS), comprised of 15,146 community-dwelling older people (42.6% men and 57.4% women) aged 60 years and older residing in the Republic of Korea. The LPOPS aimed to establish evidence-based policies for the aging population according to the Welfare of the Aged Act by monitoring trends in the health, lifestyle, and welfare status of Korean elderly people. Details of the LPOPS design are described elsewhere.^20^ The survey employed stratified two-stage cluster sampling. The primary sampling unit was based on the 2005 census frame, with secondary sampling units consisting of households with older residents. The strata consisted of 7 metropolitan and 18 provincial (urban and rural) regions with an overall response rate of 79.7%. After obtaining informed consents from study participants, trained interviewers visited the participants’ home to conduct the interviews. After excluding respondents with missing data on height, weight, and PA, data on 10,197 samples (43.3% men and 56.7% women) were used for the analysis. The institutional review board of human study reviewed and approved the study protocol. Written informed consent was obtained from all participants.

### Determination of depression and cognitive impairment

Depression was defined when diagnosed by a physician and with a score of ≥8 on the 15-item, short-form of the self-administered Korean version of Geriatric Depression Scale (GDS-K).^21^ The GDS-K and its short form were previously validated in elderly psychiatric patients.^22^

Cognitive function was assessed using the Korean version of the Mini-Mental State Examination (MMSE-KC). Mild cognitive impairment was defined as scoring more than 1.5 standard deviations below the age-, gender- and education-adjusted norms of the MMSE-KC.^23^

### Assessment of physical activity and obesity

Study participants were asked about the frequency (times/week) and duration (minutes) of PA by intensity (light, moderate, vigorous) using a short Korean version of the International Physical Activity Questionnaire (IPAQ). Each question included many types of activities that occur at work, leisure, or for transportation that lasted for at least 10 minutes. Total aerobic PA per week was estimated by summing the minutes spent in moderate PA per week and twice the minutes spent in vigorous aerobic PA per week. Aerobic PA was then categorized into three groups based on the WHO aerobic PA guidelines: 1) completely inactive (0 minutes of moderate-to-vigorous aerobic PA per week), 2) insufficiently active (1–149 minutes of moderate-to-vigorous aerobic PA per week), or 3) sufficiently active (≥150 minutes of moderate-to-vigorous PA per week). The Korean version of IPAQ short form was previously validated in a sample of Korean older adults aged 65 years or older.^24^

Height was assessed using a measuring tape with the participant standing with the back of the head, scapulae, buttocks, and heels in contact with a vertical board. Body weight was measured using a portable digital scale, after having participants remove their shoes and wear only light clothing. Body mass index (BMI) was calculated by dividing body weight by height squared (kg/m^2^). Subjects were then categorized as either underweight (≤18.5 kg/m^2^) or normal weight (>18.5–22.9 kg/m^2^) or overweight (≥23.0–24.9 kg/m^2^) or obese groups (≥25.0 kg/m^2^) based on the Asian-Pacific obesity criteria.^25^

### Covariates

The study measured social and demographic factors including age, gender, education (years), monthly income, living status (ie, alone or with family), and marital status (ie, never married or married or widowed or divorced/separated). Additionally, health behavioral factors were measured and included current smoking and alcohol consumption, number of comorbid chronic conditions, nutritional status, and self-reported health status. Smoking status was categorized as non-smoker, past smoker, or current smoker. Alcohol consumption was classified as abstinent, moderate (1–3 drinks/week), or heavy (more than the moderate level of consumption). Nutritional status was assessed using the nutrition screening initiative checklist and classified as low (0–2), moderate (3–5), or high risk (≥6) according to the checklist score.^26^ Comorbidity was defined as the number of physician-diagnosed chronic conditions (hypertension, stroke, angina, diabetes mellitus, arthritis, chronic bronchitis/emphysema, asthma, cancer, chronic renal failure, and fracture).

### Statistical analyses

Age, BMI, income, average hours of sleep per night, education, number of comorbidities, GDS, and MMSE were expressed as means and standard deviations (SDs). Total-, moderate-, and vigorous-aerobic PAs were expressed as mean and standard error of mean. Means and frequencies of sample characteristics between men and women were performed with the independent t-test and Chi-square test, respectively.

To evaluate the relationship between obesity and depression and those between PA and depression, multivariate logistic regression analyses were used, with BMI- and PA-based subgroups as the independent variables and depression as the dependent variable. Regarding the relationship between obesity and depression, the BMI-based subgroups were compared with the normal weight group as a reference. For the relationship between PA and depression, the PA-based subgroups were compared with the sufficiently active group as a reference. Additionally, the combined influence of weight status and PA for the risk of depression was analyzed. For that purpose, the insufficiently active group was collapsed into the completely inactive group, and the combined group was renamed as inactive group. Likewise, the overweight group was collapsed into the obese group, and the combined group was renamed as overweight/obese group. Then, all the subjects were reclassified in the following 6 subgroups based on PA combined with BMI levels; active and normal weight, active and underweight, active and overweight/obese, inactive and normal weight, inactive and underweight, and inactive and overweight/obese. The PA plus BMI-based subgroups were compared with the active and normal weight group as a reference.

To evaluate the possible interactions between BMI, PA, sex, and depression, a general linear model was used. We found an interaction between BMI and sex (*P* < 0.001) and between PA and sex (*P* < 0.001). Therefore, we analyzed our data separately according to gender in the multivariate logistic regression analyses. After adjustment for age, education, income, living status, marital status, smoking, alcohol consumption, nutritional status, self-reported health status, number of comorbidities, and MMSE scores, the odds ratio (OR) was calculated to study the adjusted relationship between weight status and depression, between PA and depression, and between PA combined with weight status and depression. Alpha was set at *P* = 0.05. SPSS statistical software, version 21.0 (SPSS, Inc., Armonk, NY, USA) was used to perform all statistical analyses.

## RESULTS

### Sociodemographic and health behavioral characteristics of full samples

Table 1 represents the sociodemographic characteristics and distribution of full study samples. Among the subjects, 43.3% (*N* = 4,414) were men with a mean age and BMI of 69.9 (SD, 6.4) years and BMI of 23.2 (SD, 2.9) kg/m^2^, respectively, and 56.7% (*N* = 5,783) were women with a mean age and BMI of 70.4 (SD, 6.7) years and 24.1 (SD, 3.4) kg/m^2^, respectively. The men and women had average monthly incomes of 1,160 (SD, 1,309) and 956 (SD, 1,146) USD and average night sleeping of 6.5 (SD, 1.4) and 6.2 (SD, 1.4) hours, respectively. The men and women had average comorbidity numbers of 1.4 (SD, 1.3) and 2.0 (SD, 1.5), respectively; GDS scores of 3.8 (SD, 4.0) and 4.9 (SD, 4.3); MMSE scores of 25.5 (SD, 3.4) and 23.1 (SD, 4.2); moderate PA levels of 94 (SD, 3.3) and 64 (SD, 2.4) minutes per week; vigorous PA levels of 54 (SD, 2.5) and 15 (SD, 1.1) minutes per week; and moderate-to-vigorous PA levels of 147 (SD, 4.5) and 78 (SD, 2.9) minutes per week. Between men and women, significant differences in distribution were observed in age (*P* < 0.001), education (*P* < 0.001), living status (*P* < 0.001), marital status (*P* < 0.001), smoking (*P* < 0.001), drinking (*P* < 0.001), nutritional status (*P* < 0.001), self-reported health status (*P* < 0.001), and number of comorbidities (*P* < 0.001).

**Table 1.** Baseline characteristics of full study samples

Variables	Men	Women	Total	t/χ^2^	*P*
*N*	4,414 (43.3)	5,783 (56.7)	10,197 (100)		
Age, years, mean (SD)	69.87 (6.37)	70.37 (6.72)	70.15 (6.57)	−3.798	<0.001
BMI, kg/m^2^, mean (SD)	23.16 (2.93)	24.09 (3.39)	23.67 (3.22)	−14.224	<0.001
Monthly income, USD, mean (SD)	1,160 (1,309)	956 (1,146)	1,045 (1,223)	8.385	<0.001
Night sleeping, hours/day, mean (SD)	6.49 (1.42)	6.15 (1.42)	6.29 (1.43)	12.047	<0.001
Comorbidity, number, mean (SD)	1.44 (1.33)	2.04 (1.52)	1.78 (1.48)	443.064	<0.001
GDS, scores, mean (SD)	3.78 (4.03)	4.93 (4.32)	4.43 (4.24)	−13.708	<0.001
MMSE, scores, mean (SD)	25.54 (3.41)	23.08 (4.23)	24.14 (4.08)	31.640	<0.001
Total PA, min/week, mean (SD)	147 (4.5)	78 (2.9)	108 (2.6)	15.502	<0.001
Moderate PA, min/week, mean (SD)	94 (3.3)	64 (2.4)	77 (2.0)	7.511	<0.001
Vigorous PA, min/week, mean (SD)	54 (2.5)	15 (1.1)	32 (1.2)	15.687	<0.001
**Age, years**				22.716	<0.001
60–64	990 (22.4)	1,283 (22.2)	2,273 (22.3)		
65–69	1,272 (28.8)	1,566 (27.1)	2,838 (27.8)		
70–74	1,153 (26.1)	1,395 (24.1)	2,548 (25.0)		
≥75	999 (22.6)	1,539 (26.6)	2,538 (24.9)		
**Education,**^a^ **years**				1571.585	<0.001
0–3	608 (13.8)	2,571 (44.5)	3,179 (31.2)		
4–6	1,556 (35.3)	2,126 (36.8)	3,682 (36.1)		
≥7	2,219 (50.3)	1,042 (18.0)	3,261 (32.0)		
**Living status**^b^				−29.526	<0.001
Alone	371 (8.4)	1,728 (29.9)	2,099 (20.6)		
With family	2,812 (63.7)	2,227 (38.5)	5,039 (49.4)		
**Marital status**				1818.413	<0.001
Never married	12 (0.3)	22 (0.4)	34 (0.3)		
Married	3,842 (87.0)	2,760 (47.7)	6,602 (64.7)		
Widowed	425 (9.6)	2,841 (49.1)	3,266 (32.0)		
Divorced/separated	135 (3.1)	160 (2.8)	295 (2.9)		
**Smoking**				−7.859	<0.001
Never smoker	1,147 (26.0)	5,404 (93.4)	6,551 (64.2)		
Former smoker	1,915 (43.4)	142 (2.5)	2,057 (20.2)		
Current smoker	1,352 (30.6)	237 (4.1)	1,589 (15.6)		
**Drinking frequency,**^c^ **days/week**				1618.365	<0.001
0	1,870 (42.4)	4,545 (78.6)	6,415 (62.9)		
l–3	1,821 (41.3)	1,151 (19.9)	2,972 (29.1)		
≥4	721 (16.3)	83 (1.4)	804 (7.9)		
**Nutritional status**				133.835	<0.001
Low risk	2,760 (62.5)	2,989 (51.7)	5,749 (56.4)		
Moderate risk	871 (19.7)	1,304 (22.5)	2,175 (21.3)		
High risk	783 (17.7)	1,490 (25.8)	2,273 (22.2)		
**Self-reported health status**^d^				504.118	<0.001
Very poor	287 (6.5)	532 (9.2)	819 (8.0)		
Poor	1,433 (32.5)	2,698 (46.7)	4,131 (40.5)		
Fair	988 (22.4)	1,435 (24.8)	2,423 (23.8)		
Good	1,543 (35.0)	1,049 (18.1)	2,592 (25.4)		
Very good	156 (3.3)	61 (1.1)	217 (2.1)		
**Comorbidity**				469.525	<0.001
0	1,066 (24.2)	713 (12.3)	1,779 (17.4)		
1	1,397 (31.6)	1,430 (24.7)	2,827 (27.7)		
2	1,032 (23.4)	1,498 (25.9)	2,530 (24.8)		
>3	919 (20.8)	2,142 (37.0)	3,061 (30.0)		
**BMI levels**				177.515	<0.001
Underweight	230 (5.2)	248 (4.3)	478 (4.7)		
Normal	1,899 (43.0)	1,943 (33.6)	3,842 (37.7)		
Overweight	1,186 (26.9)	1,465 (25.3)	2,651 (26.0)		
Obese	1,099 (24.9)	2,127 (36.8)	3,226 (31.6)		
**Physical activity**				122.498	<0.001
Completely inactive	2,792 (63.3)	4,451 (77.0)	7,243 (71.0)		
Insufficiently active	409 (9.3)	463 (8.0)	872 (8.6)		
Sufficiently active	1,213 (27.5)	869 (15.0)	2,082 (20.4)		
**Mild cognitive impairment**^e^				69.788	<0.001
No	3,261 (73.9)	4,668 (80.7)	7,929 (77.8)		
Yes	1,120 (25.4)	1,071 (18.5)	2,191 (21.5)		
**Depressive symptoms**				101.906	<0.001
No	3,954 (81.4)	4,213 (72.9)	7,806 (76.6)		
Yes	821 (18.6)	1,570 (27.1)	2,391 (23.4)		

BMI, body mass index; GDS, Geriatric Depression Scale; MMSE, Mini-Mental State Examination; PA, physical activity.

Depressive symptoms were defined when diagnosed by a physician or with a score of ≥8 on the 15-item, short-form of the self-administered Korean version of GDS.

^a^31 men and 44 women had no data available.

^b^1,231 men and 1,828 women had no data available.

^c^2 men and 4 women had no data available.

^d^7 men and 8 women had no data available.

^e^33 men and 44 women had no data available.

With respect to the Asian-Pacific obesity criteria, 5.2% of men were underweight, 43.3% were normal, 26.9% were overweight, and 24.9% were obese, whereas 4.3% of women were underweight, 33.6% were normal, 25.3% were overweight, and 36.8% were obese. With respect to the WHO aerobic PA guidelines, 63.3% of men were completely inactive, 9.3% were insufficiently active, and 27.5% were sufficiently active, whereas 77.0% of women were completely inactive, 8.0% were insufficiently active, and 15.0% were sufficiently active. With respect to MMSE and GDS scores, 25.4% of men and 18.5% of women suffered from MCI, whereas 18.6% of men and 27.1% of women suffered from depression. Together, the baseline characteristics of the study showed that men were physically more active (*P* < 0.001) and had lower rates of overweight/obesity (*P* < 0.001) and MCI (*P* < 0.001) and a higher rate of depression (*P* < 0.001) than women.

### Distributions in sociodemographic and health behavioral risk factors according to BMI and PA levels

Because of gender differences in distribution of BMI- or PA-based subgroups, we analyzed the baseline characteristics of men and women separately. eTable 1 and eTable 2 represent frequencies of the sociodemographic and health behavioral factors according to BMI-based subgroups.

Significant differences in distribution were observed in the variables between BMI-based subgroups in men, except marital status (*P* = 0.079), drinking (*P* = 0.149) and MCI (*P* = 0.226) (eTable 1). Overall, underweight men were older and had higher rates of sociodemographic and health behavioral risk factors than normal-weight men; a shorter education period, higher rates of current smoking and drinking, a greater number of comorbidities, and lower rates of good nutritional status and self-rated health satisfaction. In particular, underweight men had higher rate of physical inactivity (defined as completely inactive) and depression than normal, overweight, or obese men.

Likewise, significant differences in distribution were observed in the covariates between BMI-based subgroups in women, except drinking (*P* = 0.243), nutritional status (*P* = 0.087), and MCI (*P* = 0.187) (eTable 2). Underweight women were older and had higher rates of socio-demographic risk factors than normal women; a shorter education period, higher rates of never married/widowed and smoking, and a lower rate of self-rated health satisfaction. In particular, underweight women had higher rates of physical inactivity and depression than normal, overweight or obese women.

eTable 3 and eTable 4 represent frequencies of the sociodemographic and health behavioral factors according to PA-based subgroups. Significant differences in distribution were observed in the variables between PA-based subgroups in men, except smoking (*P* = 0.182) and BMI (*P* = 0.126) (eTable 3). Overall, completely inactive men were older; less educated; had higher rates of widowed and living alone, a higher number of comorbidities, and lower rates of good nutritional status and self-rated health satisfaction than sufficiently or insufficiently active men. In particular, completely inactive men had significantly higher rates of MCI and depression than sufficiently or insufficiently active men.

Likewise, significant differences in distribution were observed in the variables between PA-based subgroups in women, except smoking (*P* = 0.780), BMI (*P* = 0.743), and MCI (*P* = 0.177) (eTable 4). Overall, completely inactive women were older and less educated, drank less frequently, had a higher rate of widowed, a higher number of comorbidities, and lower rates of good nutritional status and self-rated health satisfaction than sufficiently or insufficiently active women. In particular, completely inactive women had a higher rate of depression than sufficiently or insufficiently active women.

### Odds ratios of BMI and PA levels for depression

Binary logistic regression was used to estimate the ORs of depression according to BMI and PA levels (Table 2). With respect to BMI status, underweight men and women had significantly higher risks (OR 1.77; 95% CI, 1.311–2.408 in men and OR 1.338; 95% CI, 1.010–1.772 in women) for having depression compared to normal-weight men and women, respectively. Overweight men had a significantly lower risk (OR 0.821; 95% CI, 0.673–0.993) for having depression compared to normal-weight men. However, the ORs were no longer significant between BMI-based subgroups in men (OR 1.245; 95% CI, 0.785–1.975) and women (OR 1.165; 95% CI, 0.752–1.804) after adjustments for age, education, income, night sleeping, comorbidity, living and marital status, smoking, drinking, nutrition, health status, MCI, and PA in a multivariate logistic regression model.

**Table 2.** Odds ratios for depressive symptoms according to BMI and PA levels

Variables	Men			Women			Total		
		
	OR	95% CI	*P*	OR	95% CI	*P*	OR	95% CI	*P*
**Unadjusted OR of BMI**									
Normal-weight	1			1			1		
Underweight	1.777	1.311–2.408	<0.001	1.338	1.010–1.772	0.043	1.523	1.240–1.872	<0.001
Overweight	0.821	0.673–0.993	0.043	0.879	0.754–1.026	0.102	0.876	0.778–0.987	0.030
Obese	0.850	0.700–1.032	0.010	0.977	0.851–1.121	0.977	1.008	0.903–1.125	0.893
**Adjusted OR of BMI**^a^									
Normal-weight	1			1			1		
Underweight	1.245	0.785–1.975	0.352	1.165	0.752–1.804	0.495	1.210	0.882–1.659	0.237
Overweight	1.066	0.806–1.411	0.652	0.938	0.743–1.183	0.587	0.979	0.820–1.170	0.818
Obese	1.094	0.817–1.464	0.546	1.068	0.865–1.314	0.547	1.081	0.913–1.270	0.366
**Unadjusted OR of PA**									
Sufficiently active	1			1			1		
Insufficiently active	1.016	0.693–1.489	0.935	1.253	0.926–1.695	0.144	1.231	0.975–1.55	0.081
Completely inactive	3.032	2.454–3.746	<0.001	2.519	2.067–3.069	<0.001	2.939	2.547–3.392	<0.001
**Adjusted OR of PA**^b^									
Sufficiently active	1			1			1		
Insufficiently active	0.853	0.507–1.437	0.550	1.237	0.810–1.889	0.324	1.063	0.771–1.466	0.708
Completely inactive	1.702	1.278–2.268	<0.001	1.778	1.331–2.376	<0.001	1.730	1.412–2.120	<0.001
**Unadjusted OR of PA plus BMI**									
Active + NW	1			1			1		
Active + UW	2.163	1.021–4.582	0.044	1.564	0.555–4.410	0.398	1.915	1.043–3.514	0.036
Active + OWOB	0.661	0.442–0.990	0.045	1.117	0.742–1.682	0.596	0.914	0.691–1.210	0.532
Inactive + NW	2.394	1.761–3.253	<0.001	2.689	1.888–3.831	<0.001	2.689	2.136–3.383	<0.001
Inactive + UW	3.942	2.595–5.987	<0.001	3.942	2.221–5.306	<0.001	3.827	2.842–5.514	<0.001
Inactive + OWOB	2.123	1.565–2.879	<0.001	2.123	1.760–3.524	<0.001	2.561	2.044–3.208	<0.001
**Adjusted OR of PA plus BMI**^c^									
Active + NW	1			1			1		
Active + UW	1.217	0.476–3.111	0.681	0.797	0.198–3.198	0.749	1.029	0.476–2.229	0.941
Active + OWOB	0.981	0.589–1.632	0.940	1.059	0.619–1.811	0.835	1.014	0.706–1.455	0.941
Inactive + NW	1.564	1.058–2.313	0.025	1.876	1.184–2.972	0.007	1.725	1.285–2.314	<0.001
Inactive + UW	1.952	1.108–3.439	0.021	2.122	1.176–3.827	0.012	2.036	1.363–3.040	0.001
Inactive + OWOB	1.789	1.216–2.633	0.003	1.757	1.121–2.756	0.014	1.721	1.290–2.296	<0.001

BMI, body mass index; CI, confidence interval; NW, normal weight; OR, odds ratio; OWOB, overweight and obese; PA, physical activity; UW, underweight.

^a^Adjusted ORs were statistically adjusted for age, income, education, living status, marital status, smoking, drinking, nutritional status, self-reported health status, comorbidity, mild cognitive impairment, and physical activity volume.

^b^Adjusted ORs were statistically adjusted for age, body mass index, income, education, living status, marital status, smoking, drinking, nutritional status, self-reported health status, comorbidity, and mild cognitive impairment.

^c^Adjusted ORs were statistically adjusted for age, sex, income, education, living status, marital status, smoking, drinking, nutritional status, self-reported health status, comorbidity, and mild cognitive impairment. Active: those who met WHO guidelines for PA; Inactive: those who did not meet WHO guidelines for PA.

With respect to PA levels, completely inactive men and women had significantly higher risks (OR 3.032; 95% CI, 2.454–3.746 in men and OR 2.519; 95% CI, 2.067–3.069 in women) for having depression compared to sufficiently active men and women, respectively. The ORs remained significant between PA-based subgroups in men (OR 1.702; 95% CI, 1.278–2.268) and women (OR 1.778; 95% CI, 1.331–2.376) even after adjustments for all measured covariates.

With respect to the combined influence of PA and BMI in men, the active and underweight (OR 2.163; 95% CI, 1.021–4.582), active and overweight/obese (OR 0.661; 95% CI, 0.442–0.990), inactive normal weight (OR 2.394; 95% CI, 1.761–3.253), inactive and underweight (OR 3.942; 95% CI, 2.595–5.987), and inactive and overweight/obese groups (OR 2.123; 95% CI, 1.565–2.879) had significantly higher risks for having depression compared to the active and normal weight. The ORs of the inactive and normal weight (OR 1.564; 95% CI, 1.058–2.313), inactive and underweight (OR 1.952; 95% CI, 1.108–3.439), and inactive and overweight/obese groups (OR 1.789; 95% CI, 1.216–2.633) remained significant even after adjustments for the covariates. However, the ORs of the active and underweight (OR 1.217; 95% CI, 0.476–3.111) and active and overweight/obese groups (OR 0.981; 95% CI, 0.589–1.632) were no longer significant after adjustments for the covariates.

With respect to the combined influence of PA and BMI in women, the inactive and normal weight (OR 2.689; 95% CI, 1.888–3.831), inactive and underweight (OR 3.942; 95% CI, 2.221–5.306), and inactive and overweight/obese groups (OR 2.123; 95% CI, 1.760–3.524) has significantly higher risks of having depression compared to the active and normal weight. The ORs of the inactive and normal weight (OR 1.876; 95% CI, 1.184–2.972), inactive and underweight (OR 2.122; 95% CI, 1.176–3.827), and inactive and overweight/obese groups (OR 1.757; 95% CI, 1.121–2.756) remained significant even after adjustments for the covariates. However, the active and underweight (OR 1.564; 95% CI, 0.555–4.410) and active and overweight/obese groups (OR 0.742; 95% CI, 0.742–1.682) had no significant differences in the risks of having depression compared to the active and normal weight.

## DISCUSSION

Depression is a major public health problem, with a 16% lifetime incidence of diagnosis in the general population.^27^ Because depression, obesity, and physical inactivity carry the increased risk of cardio-metabolic disorders such as coronary heart disease and type 2 diabetes, exploring the relationships between these conditions has received increased attention in the literature. This population-based, cross-sectional study demonstrates the importance of a healthy lifestyle, characterized by meeting the aerobic PA guidelines and maintaining a healthy body weight, in lowering the risk of late life depression in Korean adults.

With respect to the baseline characteristics, we found that those who were underweight or completely inactive had poorer sociodemographic and health behavioral circumstances in conjunction with increased risk of depression, as compared to those who were normal weight or sufficiently active. These results are in accordance with those of previous studies reporting that age, female gender, separation or divorce, low education, and low income were identified as sociodemographic risk factors in explaining variability in the prevalence of depression.^28^ Previous studies also reported that health behavioral factors such as smoking, drinking, nutritional status, self-reported health satisfaction were important risk factors for the mental disorders.^28^^,^^29^

With respect to BMI, we found that being underweight was significantly associated with higher rates of the sociodemographic and health behavioral risk factors as well as higher risk of late life depression in both men and women, with no such associations between obesity and depression. In this study, we found that underweight individuals had significantly higher risk scores than normal weight individuals (*P* = 0.040). The negative impact of being underweight on depression observed in this study is consistent with the previous findings from Korean^11^^,^^14^ and Chinese^30^ older population-based studies. Furthermore, we analyzed the effect of being underweight on depression by entering the sociodemographic and health behavioral factors as covariates into a multivariate regression model and found that the OR of being underweight for having depression was no longer significant in men, women, or the full sample after controlling for the covariates. Consequently, current findings suggest that both sociodemographic and health behavioral risk factors can act as important modulators in determining the association between underweight and/obese and late life depression.

With respect to PA levels, we found that being completely inactive (or not meeting the WHO guidelines for weekly PA) was significantly associated with higher rates of the sociodemographic and health behavioral risk factors as well as higher risk of depression in both men and women. In particular, a multivariate logistic regression analysis showed that the risk of not meeting the aerobic PA guidelines for having depression remained significant even after adjustments for all measured covariates, suggesting the independent role of PA as a predictor of late life depression.

With respect to the combined effect of PA and BMI, we found that in both men and women, the risk of depression was highest in the inactive and underweight group, followed by the inactive and overweight/obese and inactive and normal weight in order, suggesting an additive influence of being physical inactive with underweight as well as overweight/obesity for the risk of late life depression in this study population.

Current findings are consistent with those from previous studies reporting decreased PA as a significant determinant of depression in cross-sectional^16^ and longitudinal studies^18^^,^^19^ as well as in a hybrid study involving healthy older women.^31^ However, some studies reported a bi-directional relationship between PA and depression. Lampinen et al^32^ confirmed in an 8 year-follow-up that elderly persons who reduced activities had higher risks of depressive symptoms compared to those who increased or maintained PA. In analyzing 1,920 elderly individuals for 6 years, van Gool et al^33^ showed that those who became depressive were more likely to lead a sedentary lifestyle than those without depression. Together, the previous findings support the current study findings regarding PA as a preventive and/or therapeutic measure for late life depression. Yet, the nature of cross-sectional studies, including the current one, makes it difficult to draw a definitive conclusion about the association between PA and depression in a cause-and-effect manner.

There are several possible mechanisms to explain PA-induced antidepressant effect. First, PA provides an antidepressant effect via an endorphin release of neurotransmitters (ie, serotonin, dopamine, and norepinephrine).^34^ Second, PA is known to serve a distraction from worries and depressing symptoms and thoughts,^35^ perhaps due to an increased release of β-endorphins following exercise.^36^ Third, the enhancement of self-efficacy through regular PA may be another way in which exercise exerts its antidepressant effects.^37^ Lastly, pro-inflammatory cytokines and reductions of growth factors are inversely related to the severity of depression.^38^ Thus, PA-induced antidepressant effect may involve the upregulation of anti-inflammatory cytokines^39^ and growth factors such as brain-derived neurotrophic factor, insulin-like growth factor, and vascular endothelial growth factors.^40^

This study has several limitations. First, the nature of the cross-sectional studies, including the current one, and potentially bi-directional relationships between the risk factors and mental disorders make it difficult to draw a definitive conclusion about the association between the risk factors and mental disorders in a cause-and-effect manner. Second, BMI is widely used as an indicator of nutritional status due to its strong correlation with body mass and weak correlation with stature.^41^ Among older adults, however, the use of the BMI is problematic in light of the decrease in stature, accumulation of fatty tissues, reductions in lean body mass, and decrease in the amount of body fluids. The use of the BMI in elderly persons is further complicated by the frequent presence of diseases and the lack of specific cutoff points for this age group.^42^^,^^43^ Consequently, the association between obesity and depression should be confirmed in a future study using a more reliable indicator of adiposity in older adults. Third, functional biomarkers of depression, such as growth factors, inflammatory cytokines, endocrine factors and metabolic markers, which might influence the associations between weight status, PA, and late life depression, were not available in this study. Fourth, no clinical history of any other mental disorders not accounted for in this study was obtained. Therefore, it cannot be deduced whether existing mental disorders are responsible for the associations between PA, obesity, and late life depression.

In conclusion, we found that compared to those who met the aerobic PA guidelines, those who not met them had a higher rate of late life depression in conjunction with higher rates of sociodemographic and health behavioral risk factors in the same population. The OR of being completely inactive (ie, not meeting the aerobic PA guidelines at all) for having late life depression remained significant even after adjustments for the covariates. Furthermore, the combined analysis of PA and BMI levels for having depression showed that being inactive with underweight and/or overweight and obese was the highest risk factor of depression in this study population, suggesting a critical role of being physically active and maintaining normal body weight as a preventive/therapeutic measure for mental health problems, including late life depression in Korean older adults.

## APPENDIX A. SUPPLEMENTARY DATA

eTable 1. Baseline characteristics of men study population according to BMI levels

eTable 2. Baseline characteristics of women study population according to BMI levels

eTable 3. Baseline characteristics of men study population according to weekly physical activity levels

eTable 4. Baseline characteristics of women study population according to weekly physical activity levels
