# Supplementary material for: Weight Status, Physical Activity, and Depression in Korean Older Adults
Source: J Epidemiol. 2018 Jun 5;28(6):292–9. doi: 10.2188/jea.JE20170083 (PMC5976873; doi:10.2188/jea.JE20170083)
Supplement: Supplementary file 1 [file je-28-292-s001.pdf]

eTable 1. Baseline characteristics of men study population according to BMI levels

Variables	UW<br>N (%)	NW<br>N (%)	OW<br>N (%)	OB<br>N (%)	t/χ^2	P
**Age, years**					82.611	<0.001
60–64	31 (3.1)	375 (37.9)	299 (30.2)	285 (28.8)		
65–69	58 (4.6)	504 (39.6)	346 (27.2)	364 (28.6)		
70–74	57 (4.9)	522 (45.3)	302 (26.2)	272 (23.6)		
≥75	84 (8.4)	498 (49.8)	239 (23.9)	178 (17.8)		
**Education, years**					34.211	<0.001
0–3	45 (19.6)	283 (14.9)	147 (12.4)	133 (12.1)		
4–6	100 (43.5)	690 (36.3)	404 (34.1)	362 (32.9)		
≥7	84(36.5)	910 (47.9)	626 (52.8)	599 (54.5)		
**Living status**					9.545	0.023
Alone	21 (9.1)	188 (9.9)	84 (7.1)	78 (7.1)		
With family	146 (63.5)	1,200 (63.2)	765 (64.5)	701 (63.8)		
**Marital status**					6.783	0.079
Never married	1 (0.4)	3 (0.2)	6 (0.5)	2 (0.2)		
Married	195 (84.8)	1,629 (85.7)	1,047 (88.3)	971 (88.4)		
Widowed	25 (10.9)	207 (10.9)	97 (8.2)	96 (8.7)		
Divorced/separated	9 (3.9)	60 (3.2)	36 (3.0)	30 (2.7)		
**Smoking**					63.185	<0.001
Never smoker	42 (18.3)	446 (23.5)	316 (26.6)	343 (31.2)		
Former smoker	86 (37.4)	790 (41.6)	516 (43.5)	523 (47.6)		
Current smoker	102 (44.3)	663 (34.9)	354 (29.8)	233 (21.2)		
**Drinking frequency, days/week**					5.333	0.149
0	113 (49.1)	839 (44.2)	494 (41.7)	424 (38.6)		
l–3	70 (30.4)	738 (38.9)	514 (43.3)	499 (45.4)		
≥4	47 (20.4)	321 (16.9)	177 (14.9)	176 (16.0)		
**Nutritional status**					28.372	<0.001
Low risk	112 (48.7)	1,158 (61.0)	786 (64.1)	704 (64.1)		
Moderate risk	66 (28.7)	365 (19.2)	210 (20.9)	230 (20.9)		
High risk	52 (22.6)	376 (19.8)	190 (16.0)	165 (15.0)		
**Self-reported health status**					61.896	<0.001
Very poor	32 (13.9)	127 (6.7)	62 (5.2)	66 (6.0)		

Poor	94 (40.9)	643 (33.9)	335 (28.2)	361 (32.8)		
Fair	56 (24.3)	433 (22.8)	270 (22.8)	229 (20.8)		
Good	44 (19.1)	644 (33.9)	460 (38.8)	395 (35.9)		
Very good	4 (1.7)	48 (2.5)	58 (4.9)	46 (4.2)		
**Comorbidity**					34.911	<0.001
0	58 (25.2)	505 (26.6)	299 (25.2)	204 (18.6)		
1	68 (29.6)	617 (32.5)	365 (30.8)	347 (31.6)		
2	51 (22.2)	433 (22.8)	290 (24.5)	258 (27.5)		
≥3	53 (23.0)	344 (18.1)	232 (19.6)	290 (26.4)		
**Physical activity**					9.488	0.023
Completely inactive	161 (70.0)	1,220 (64.2)	718 (60.5)	693 (63.1)		
Insufficiently active	172 (9.1)	172 (9.1)	119 (10.0)	97 (8.8)		
Sufficiently active	507 (26.7)	507 (26.7)	349 (29.4)	309 (28.1)		
**Mild cognitive impairment**					4.355	0.226
No	166 (72.2)	1,380 (72.7)	901 (76.0)	814 (74.1)		
Yes	63 (27.4)	501 (26.4)	276 (23.3)	280 (25.5)		
**Depressive symptoms**					25.795	<0.001
No	161 (70.0)	1,530 (80.6)	990 (83.5)	912 (83.0)		
Yes	69 (30.0)	369 (19.4)	196 (16.5)	187 (17.0)		

BMI, body mass index; UW, underweight; NW, normal weight; OW, overweight; OB, obese.

eTable 2. Baseline characteristics of women study population according to BMI levels

Variables	UW N (%)	NW N (%)	OW N (%)	OB N (%)	t/χ^2	P
**Age, years**					134.483	<0.001
60–64	31 (2.4)	362 (28.2)	350 (27.3)	540 (42.1)		
65–69	43 (2.7)	473 (30.2)	412 (26.3)	638 (40.7)		
70–74	52 (3.7)	491 (35.2)	353 (25.3)	499 (35.8)		
≥75	122 (7.9)	617 (40.1)	350 (22.7)	450 (29.2)		
**Education, years**					42.805	<0.001
0–3	141 (56.9)	938 (48.3)	583 (39.8)	909 (42.7)		
4–6	79 (31.9)	660 (34.0)	564 (38.5)	823 (38.7)		
≥7	26 (10.5)	332 (17.1)	304 (20.8)	380 (17.9)		
**Living status**					9.528	0.023
Alone	83 (33.5)	619 (31.9)	412 (28.1)	614 (28.9)		
With family	81 (32.7)	726 (37.4)	587 (40.1)	833 (39.2)		
**Marital status**					16.623	0.001
Never married	4 (1.6)	5 (0.3)	7 (0.3)	8 (0.4)		
Married	84 (33.5)	891 (45.9)	737 (50.3)	1,049 (49.3)		
Widowed	156 (62.9)	995 (51.2)	868 (46.8)	1,004 (47.2)		
Divorced/separated	5 (2.0)	52 (2.7)	37 (2.5)	66 (3.1)		
**Smoking**					32.497	<0.001
Never smoker	211 (85.1)	1,789 (92.1)	1,380 (94.2)	2,024 (95.2)		
Former smoker	6 (2.4)	50 (2.6)	31 (2.1)	55 (2.6)		
Current smoker	31 (12.5)	104 (5.4)	54 (3.7)	48 (2.3)		
**Drinking frequency, day/week**					4.181	0.243
0	206 (83.1)	1,536 (79.1)	1,132 (77.3)	1,671 (78.6)		
l–3	36 (14.5)	372 (19.1)	312 (21.3)	431 (20.3)		
≥4	6 (2.4)	33 (1.7)	20 (1.4)	24 (1.1)		
**Nutritional status**					6.577	0.087
Low risk	118 (47.6)	978 (50.3)	758 (51.7)	1,135 (53.4)		
Moderate risk	58 (23.4)	438 (22.5)	336 (22.9)	472 (22.2)		
High risk	72 (29.0)	527 (27.1)	371 (25.3)	520 (24.4)		
**Self-reported health status**					13.324	0.004
Very poor	25 (10.1)	198 (10.2)	110 (7.5)	199 (9.4)		

Poor	127 (51.2)	865 (44.5)	666 (45.5)	1,040 (48.9)		
Fair	58 (23.4)	503 (25.9)	388 (26.5)	486 (22.8)		
Good	38 (15.3)	352 (18.1)	278 (19.0)	381 (17.9)		
Very good	0	20 (1.0)	23 (1.6)	18 (0.8)		
**Comorbidity**					52.475	<0.001
0	40 (16.1)	295 (15.2)	181 (12.4)	197 (9.3)		
1	74 (29.8)	508 (26.1)	359 (24.5)	489 (23.0)		
2	61 (24.6)	483 (24.9)	384 (26.2)	570 (26.8)		
≥3	73 (29.4)	657 (33.8)	541 (36.9)	871 (40.9)		
**Physical activity**					20.613	<0.001
Completely inactive	210 (84.7)	1,503 (77.4)	1,074 (73.3)	1,664 (78.2)		
Insufficiently active	13 (5.2)	157 (8.1)	139 (9.5)	154 (7.2)		
Sufficiently active	25 (10.1)	283 (14.6)	252 (17.2)	309 (14.5)		
**Mild cognitive impairment**					4.801	0.187
No	197 (79.4)	1,576 (81.1)	1,155 (78.8)	1,740 (81.8)		
Yes	49 (19.8)	354 (18.2)	296 (20.2)	372 (17.5)		
**Depressive symptoms**					8.806	0.032
No	164 (66.1)	1,405 (72.3)	1,096 (74.8)	1,548 (72.8)		
Yes	84 (33.9)	538 (27.7)	369 (25.2)	579 (27.2)		

BMI, body mass index; UW, underweight; NW, normal weight; OW, overweight; OB, obese.

eTable 3. Baseline characteristics of men study population according to weekly physical activity levels

Variables	Completely inactive, N (%)	Insufficiently active, N (%)	Sufficiently active, N (%)	t/χ^2	P
**Age, years**				64.365	<0.001
60–64	569 (20.4)	109 (26.7)	312 (25.7)		
65–69	757 (27.1)	129 (31.5)	386 (31.8)		
70–74	718 (25.7)	100 (24.4)	335 (27.6)		
≥75	748 (26.8)	71 (17.4)	180 (14.8)		
**Education, years**				28.659	<0.001
0-3	431 (15.4)	42 (10.3)	135 (11.1)		
4–6	1,013 (36.3)	137 (33.5)	406 (33.5)		
≥7	1,325 (47.5)	227 (55.5)	667 (55.0)		
**Living status**				21.235	<0.001
Alone	270 (9.7)	31(7.6)	70 (5.8)		
With family	1,710 (61.2)	273 (66.7)	829 (68.3)		
**Marital status**				47.444	<0.001
Never married	6 (0.2)	0	6 (0.5)		
Married	2,364 (84.7)	3,721 (90.7)	1,107 (91.3)		
Widowed	333 (11.9)	24 (5.9)	68 (5.6)		
Divorced/separated	89 (3.2)	14 (3.4)	32 (2.6)		
**Smoking**				3.412	0.182
Never smoker	708 (25.4)	115 (28.1)	324 (26.7)		
Former smoker	1,203 (43.1)	186 (45.5)	363 (29.9)		
Current smoker	881 (31.6)	108 (26.4)	526 (43.4)		
**Drinking frequency, day/week**				25.564	<0.001
0	1,270 (45.5)	151 (36.9)	449 (37.0)		
l–3	1,085 (38.9)	203 (49.6)	533 (43.9)		
≥4	436 (15.6)	55 (13.4)	230 (19.0)		
**Nutritional status**				56.901	<0.001
Low risk	1,636 (58.6)	281 (68.7)	843 (69.5)		
Moderate risk	581 (20.8)	79 (19.3)	211 (17.4)		
High risk	575 (20.6)	49 (12.0)	159 (13.1)		
**Self-reported health status**				204.498	<0.001
Very poor	214 (8.6)	12 (2.9)	34 (2.8)		

Poor	1,062 (38.0)	99 (24.2)	272 (22.4)		
Fair	581 (20.8)	123 (30.1)	284(23.4)		
Good	828 (29.7)	162 (39.6)	553 (45.6)		
Very good	75 (2.7)	12 (2.9)	69 (5.7)		
**Comorbidity**				37.524	<0.001
0	606 (21.7)	119 (29.1)	341 (28.1)		
1	873 (31.3)	130 (31.8)	394 (32.5)		
2	666 (23.9)	94 (23.0)	272 (22.4)		
≥3	647 (23.2)	66 (16.1)	206 (17.0)		
**BMI levels**				4.146	0.126
Underweight	161 (5.8)	21 (5.1)	48 (4.0)		
Normal	1,220 (43.7)	172 (42.1)	507 (41.8)		
Overweight	718 (25.7)	119 (29.1)	349 (28.8)		
Obese	693 (24.8)	97 (23.7)	309 (25.5)		
**Mild cognitive impairment**				6.395	0.041
No	2,026 (72.6)	308 (75.3)	927 (76.4)		
Yes	743 (26.6)	96 (23.5)	281 (23.2)		
**Depressive symptoms**				142.302	<0.001
No	2,124 (76.1)	370 (90.5)	1,099 (69.5)		
Yes	668 (23.9)	39 (9.5)	114 (9.4)		

BMI, body mass index.

eTable 4. Baseline characteristics of women study population according to weekly physical activity levels

Variables	Completely inactive, N (%)	Insufficiently active, N (%)	Sufficiently active, N (%)	t/χ^2	P
**Age, years**				136.626	<0.001
60–64	877 (19.7)	133 (28.7)	273 (31.4)		
65–69	1,167 (26.2)	116 (25.1)	283 (32.6)		
70–74	1,083 (24.3)	118 (25.5)	194 (22.3)		
≥75	1,324 (29.7)	96 (20.7)	119 (13.7)		
**Education, years**				77.636	<0.001
0–3	2,013 (47.2)	172 (37.1)	296 (34.1)		
4–6	1,594 (35.8)	183 (39.5)	349 (40.2)		
≥7	718 (16.1)	106 (22.9)	218 (25.1)		
**Living status**				44.334	<0.001
With family	1,580 (35.5)	215 (46.4)	432(49.7)		
Alone	1,383 (31.1)	129 (27.9)	216 (24.9)		
**Marital status**				86.722	<0.001
Never married	19 (0.4)	1 (0.2)	2 (0.2)		
Married	1,980 (44.5)	262 (56.6)	518 (59.6)		
Widowed	2,331 (52.4)	189 (40.8)	321 (36.9)		
Divorced/separated	121 (2.7)	11 (2.4)	28 (3.2)		
**Smoking**				0.498	0.780
Never smoker	4,149 (93.2)	429 (92.7)	826 (95.1)		
Former smoker	115 (2.6)	13 (2.8)	29 (3.3)		
Current smoker	187 (4.2)	21 (4.5)	14 91.6)		
**Drinking frequency, day/week**				21.624	<0.001
0	3,557 (79.9)	350 (75.6)	638 (73.4)		
l–3	832 (18.7)	104 (22.5)	215 (24.7)		
≥4	58 (1.3)	9 (1.9)	16 (1.8)		
**Nutritional status**				53.588	<0.001
Low risk	2,184 (49.1)	274 (59.2)	531 (61.1)		
Moderate risk	1,045 (23.5)	90 (19.4)	169 (19.4)		
High risk	1,222 (27.5)	99 (21.4)	169 (19.4)		
**Self-reported health status**				173.360	<0.001
Very poor	475 (10.7)	24 (5.2)	33 (3.8)		

Poor	2,183 (49.0)	191 (41.3)	324 (37.2)		
Fair	1,075 (24.2)	129 (27.9)	231 (26.6)		
Good	675 (15.2)	113 (24.4)	261 (30.0)		
Very good	36 (0.8)	6 (1.3)	19 (2.2)		
**Comorbidity**				21.707	<0.001
0	505 (11.3)	61 (13.2)	147 (16.9)		
1	1,095 (24.6)	114 (24.6)	221 (25.4)		
2	1,186 (26.6)	101 (21.8)	211 (24.3)		
≥3	1,665 (37.4)	187 (40.4)	290 (33.4)		
**BMI levels**				0.595	0.743
Underweight	210 (4.7)	13 (2.8)	25 (2.9)		
Normal	1,503 (33.8)	157 (33.9)	283 (32.6)		
Overweight	1,074 (24.1)	139 (30.0)	252 (29.0)		
Obese	1,664 (37.4)	154 (33.3)	309 (35.6)		
**Mild cognitive impairment**				3.468	0.177
No	3,368 (80.2)	382 (82.5)	718 (82.6)		
Yes	847 (19.0)	79 (17.1)	145 (16.7)		
**Depressive symptoms**				111.833	<0.001
No	3,093 (69.5)	380 (82.1)	740 (85.2)		
Yes	1,358 (30.5)	83 (17.9)	129 (14.8)		

BMI, body mass index.
